# Mechanisms of Abscisic Acid-Mediated Drought Stress Responses in Plants

**DOI:** 10.3390/ijms23031084

**Published:** 2022-01-19

**Authors:** Mehtab Muhammad Aslam, Muhammad Waseem, Bello Hassan Jakada, Eyalira Jacob Okal, Zuliang Lei, Hafiz Sohaib Ahmad Saqib, Wei Yuan, Weifeng Xu, Qian Zhang

**Affiliations:** 1 Joint International Research Laboratory of Water and Nutrient in Crop and College of Resources and Environment, Fujian Agriculture and Forestry University, Fuzhou 350002, China; mehtabmuhammadaslam@yahoo.com (M.M.A.); 1200525014@fafu.edu.cn (Z.L.); wfxu@fafu.edu.cn (W.X.); 2 College of Agriculture, Yangzhou University, Yangzhou 225009, China; 3 Department of Botany, University of Narowal, Narowal 51600, Pakistan; m.waseem.botanist@gmail.com; 4 College of Horticulture, Hainan University, Haikou 570100, China; 5 Key Laboratory of Genetics, Breeding and Multiple Utilization of Crops, College of Life Science, Fujian Agriculture and Forestry University, Ministry of Education, Fuzhou 350002, China; bellojakada@gmail.com; 6 Center for Mountain Futures, Kunming Institute of Botany, Chinese Academy of Sciences, Kunming 650201, China; eyalirajac@gmail.com; 7 Guangdong Provincial Key Laboratory of Marine Biology, College of Science, Shantou University, Shantou 515063, China; sohaibsaqib@gmail.com

**Keywords:** ABA, drought, metabolites, signaling, crop breeding

## Abstract

Drought is one of the major constraints to rain-fed agricultural production, especially under climate change conditions. Plants evolved an array of adaptive strategies that perceive stress stimuli and respond to these stress signals through specific mechanisms. Abscisic acid (ABA) is a premier signal for plants to respond to drought and plays a critical role in plant growth and development. ABA triggers a variety of physiological processes such as stomatal closure, root system modulation, organizing soil microbial communities, activation of transcriptional and post-transcriptional gene expression, and metabolic alterations. Thus, understanding the mechanisms of ABA-mediated drought responses in plants is critical for ensuring crop yield and global food security. In this review, we highlighted how plants adjust ABA perception, transcriptional levels of ABA- and drought-related genes, and regulation of metabolic pathways to alter drought stress responses at both cellular and the whole plant level. Understanding the synergetic role of drought and ABA will strengthen our knowledge to develop stress-resilient crops through integrated advanced biotechnology approaches. This review will elaborate on ABA-mediated drought responses at genetic, biochemical, and molecular levels in plants, which is critical for advancement in stress biology research.

## 1. Introduction

Drought stress reduces soil water content which restricts water uptake by the plant root thereby limiting plant growth and productivity [1]. Plants have evolved a wide range of morpho-physiological, metabolic, and molecular mechanisms to resist long- or short-term responses to drought stress [2]. Phytohormones are important plant growth regulators and mediators of environmental stresses such as drought which adversely influence crop yield and pose threats to global food security [2]. To cope with drought stress, potent and novel approaches should be introduced, and phytohormone engineering could be a method of choice for sustainable crop production and breeding programs. In the last decade, the interest to understand the spatiotemporal changes of ABA to modulate plant responses is growing [3]. Abscisic acid (ABA) is critical for plant development and can redesign various physiological and biochemical signal transduction cascades in plants to cope with environmental stresses particularly drought [4,5]. Additionally, ABA plays a critical role in biomolecules biosynthesis, senescence, seed germination, stomatal closure, and root architecture modification [6,7].

**Figure 1:**
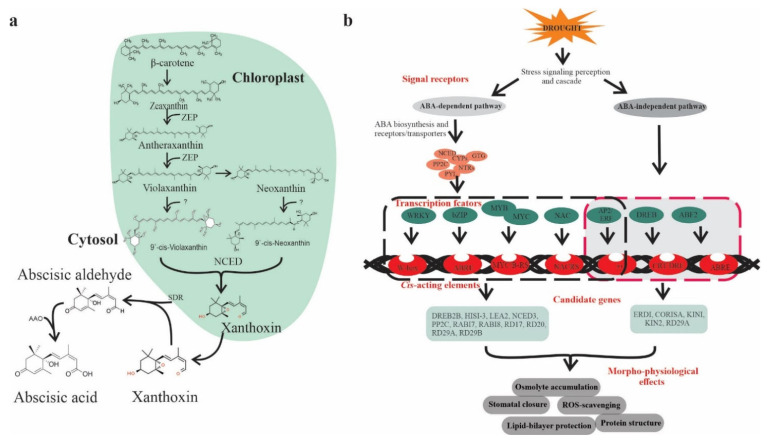
ABA biosynthesis and ABA-mediated drought-responsive pathways in plants. (**a**) Scheme of ABA biosynthesis. The precursors of ABA, *β*-carotene undergoes a series of oxidative reactions in the plastids and each step is catalyzed by specific enzyme such as ZEP (zeaxanthin epoxidase) or NCED (9-*cis*-epoxycarotenoid dioxygenase). The derived xanthoxin is exported to the cytosol and converted into ABA through an oxidation reaction mediated by AAO (aldehyde oxidase) and SDR (alcohol dehydrogenase/reductase), (**b**) ABA-dependent and -independent signaling pathways in the plant, which consists of several core components including ABA receptors and regulators. The ABA-dependent and -independent pathways are indicated by black and red arrows, respectively. Transcription factors (TFs) include bZIPs, MYB/MYC2, NAC (RD26), and WRKY bind to their corresponding *cis*-acting elements W-box, ABRE, MYB, MYC, DREB2, AREB/ABF, and NACRs.

ABA is classified as an isoprenoid molecule, synthesized from carotenoids (C40) derivative of isopentenyl diphosphate (IPP) through the methylerythritol phosphate (MEP) pathway in plastids [8]. The synthesis of ABA undergoes a series of steps, and each step is catalyzed by a specific enzyme. The conversion of zeaxanthin to all trans-violaxanthin is the first step in ABA biosynthesis occurring in the plastid. This cyclic hydroxylation of epoxycarotenoids to all-xanthin is catalyzed by *zeaxanthin epoxidase* (ZEP) through an intermediate antheraxanthin. In the next step, cis-isomerization of all trans-violaxanthin to violaxanthin or cisneoxanthin through an unknown enzymatic reaction. After that, 9-cis-epoxycarotenoid dioxygenase (NCED) enzymes split the cis-isomers of violaxanthin and neoxanthin to generate a C15 intermediate product called xanthoxin, finally exported to cytosol. In the cytosol, xanthoxin is converted into ABA through two enzymatic reactions. Next, xanthoxin is first converted to an abscisic aldehyde catalyzed by short-chain alcohol dehydrogenase/reductase (SDR). Finally, the oxidation of abscisic aldehyde to ABA by *aldehyde oxidase* (AAO) (Figure 1a) [9].

Plants show a significant increase in ABA levels under drought stress, changes in expression of genes, and induction of ABA biosynthesis enzymes corresponding to mRNA level lead to enhanced ABA accumulation [10]. The transcript abundances of several ABA biosynthesis genes, such as *ZEP/ABA1, AAO3, 9-cis-epoxycarotenoid dioxygenase (NCED3),* and *molybdenum cofactor sulfurase (MCSU/LOS5/ABA3)*, has been upregulated through an ABA-dependent or ABA-independent pathway [11] assisted by binding factors such as ABF, MYC MYB, NAC, ERF, bZIP, and DREB/CBF transcription factors (TFs) (Figure 1b) [12].

ABA is a prime mediator of drought [13] and plays an important role in regulating plant growth, development, and responses to several environmental stresses [14]. Under drought conditions, ABA-mediated stomatal conductance prevents transpiration water loss [10]. Zhang et al. [15] found that multidrug and toxic compound extrusion (MATE) transporter family, *detoxification efflux carrier* (*AtDTX50*), participate in ABA transport. ABA receptors such as PYRABACTIN RESISTANCE (PYR), or regulatory component of ABA receptor (RCAR) enhanced ABA responses and confer drought tolerance in Arabidopsis [16]. Similarly, ABA responsive-element binding protein (ABP9) a member of the bZIP family (Figure 1) improves photosynthetic capacity under drought [17]. Histone acetylation has been reported to be critical in ABA-mediated gene regulation to acclimatize plants to drought [18]. It has been shown that mitogen-activated protein kinase (MAPK) signaling cascade plays a critical role in ABA-mediated drought regulation at transcription and proteome level in various plant species including rice, maize, and Arabidopsis [19,20]. Altogether, ABA is a pivotal hormone governing plant responses to drought through complex molecular signaling mechanisms. Therefore, exploration of ABA regulators could assist in developing drought-tolerant crops through breeding programs.

The roles of ABA have been extensively studied through the implementation of molecular and genetics approaches, which enable the development of drought-resilient crops. This review is an attempt to underline the role of ABA in drought adaption, avoidance, in different biochemical and physiological responses. Moreover, we also highlighted a concise overview of the molecular mechanism of ABA actions and ABA crosstalk with other hormones in regulating drought stress responses.

## 2. ABA: A Key Player under Drought

Abscisic acid is of prime importance due to its stress-related responses and its involvement in various plant growth processes, making it possible to adapt to drought conditions. Upon drought stress, ABA-mediated stomatal closure reduces water loss by decreasing transpiration rate. Moreover, ABA progressively increases hydraulic conductivity and stimulates root cell elongation, enabling plants recovery from water-limited conditions [21]. Recent advancements in plant genomics accelerated the identification and functional characterization of ABA-dependent candidate genes responsive to drought. For instance, Zhang et al. [15] found that the MATE transporter gene, *AtDTX50*, is involved in ABA efflux, while mutants of *dtx50* show enhanced tolerance to drought with reduced stomatal conductance relative to WT plants. It is widely acknowledged that ABA binds to pyrabactin-resistance 1/pyrabactin resistance like/regulatory component of aba receptor (PYR/PYL/RCAR) receptors, the initial step of the core ABA signaling pathway, concerning previously characterized protein phosphatases 2C (PP2Cs) and sucrose nonfermenting related kinases 2 (SnRK2s) (Figure 1b) [22,23,24]. The PYR/PYL/RCAR) proteins are reported to be involved in improving drought tolerance in many species such as *Arabidopsis*, tomato, and rice [25,26,27,28].

ABA has also been reported to regulate calcium-dependent protein kinases (CPK) signaling by inducing *CPK6* expression under drought stress. CPKs interact and phosphorylates some core ABA-related TFs, ABFs/AREBs (ABA-responsive element-binding factors) enhancing their transcriptional activities [29]. Similarly, transgenic plants overexpressing ZEP confers tolerance to stresses such as drought [30]. Overexpression of *OsbZIP72* showed increased expression of ABA-responsive gene *LEAs (late embryogenesis abundant genes*) and improved drought resistance in rice, which may be useful for the engineering of drought-resilient crops [31]. Arabidopsis plants overexpressing *ABCG25* showed reduced water loss under drought by limiting evapotranspiration. Likewise, mutants of *AtABCG40* exhibited more sensitivity to drought [32], indicating the prime importance of ABA-related genes in regulating ABA responses to drought conditions.

The ABA hormone has mainly been associated with the regulation of water deficiency in plants. A plethora of studies have shown the critical roles of ABA in regulating genes expression, proteins, and enzymatic activities involved in plant cell dehydration tolerance [33,34]. For instance, the ABA levels were exponentially elevated in *Arabidopsis*, wheat, rice, tomato, soybean, maize, and sesame under drought [35,36]. Similarly, Wang et al. [37] and Baek et al. [38] demonstrated how multiple genes regulate ABA-mediated drought responses in *Arabidopsis*, *Vigna. radiata*, and *V. angularis*. These findings suggest that ABA-mediated drought tolerance is required for plants to fully respond to drought stress.

## 3. ABA-Mediated Drought Responses through Physio-Biochemical Alteration

Plants have evolved distinct adaptive mechanisms to survive and minimize the adverse effect of drought stress [39]. Reactive oxygen species (ROS) serve as a signal molecule that regulates plant responses to stresses. Upon drought, plants synthesize an array of secondary metabolites (SMs) assisting plant survival [40]. ABA is able to synchronize a wide range of functions in plants, facilitating to overcome drought stress [4]. Therefore, to tackle water limitations, dynamic and novel strategies should be formulated and engineered including ROS and SMs as an adaptive strategy to maintain plant growth and productivity.

### 3.1. ROS Scavenging System

Drought and ABA have an intricate relationship [41] that triggers various downstream responses to plant assisting adaption to drought in an ABA-dependent manner [14]. Drought may alter the metabolic and cellular redox status of plants that influence the cellular susceptibility to ABA accumulation [42] suggested the link between metabolic status and ABA signaling [43]. ABA is an indicator of soil water deficit and endogenous ABA concentration rapidly increases to initiate stomatal closure in the plant [44]. Previous studies have also been demonstrated that drought escape induced by water stress depends on ABA. For instance, ABA could improve the plant ability to scavenge ROS by activating antioxidant enzymes [45] such as SOD (superoxide dismutase), POD (peroxidase), CAT (catalase), APX (ascorbate peroxidase), and GR (glutathione reductase) in wheat seedlings under drought, thus regulating the osmotic adjustment, reducing oxidative damage, and improving the conductivity of roots by inducing aquaporin gene expression [45,46,47]. Kwak et al. [48] showed that ABA activates H_2_O_2_ biosynthesis in stomata guard cells via a membrane-bound NADPH oxidase causing stomata closure by activating plasma membrane Ca^2+^ channels [49].

### 3.2. Primary Metabolism

Land plants synthesize diverse primary metabolites (PMs) having higher medicinal and nutritional value which are essential for survival [50]. In general, PMs function in protein–disulfide linkage, redox regulation, methylation reactions, including DNA methylation, mRNA capping, synthesis of phosphatidylcholine, and synthesis of polyamines [51]. Primary metabolites and their associated metabolic genes are considered pivotal factors that contribute to drought tolerance via the involvement of different metabolic pathways [52]. To date, in planta, an estimate of 200,000 metabolites are reported [53]. Among those carbohydrates, nucleosides/nucleotides, and sulfur-containing metabolites were mainly induced by ABA [54]. The major pathways responsible for PMs are glycolysis, the TCA cycle, pentose phosphate pathway, shikimate pathway, aliphatic, and aromatic amino acids which produce secondary metabolites (SMs). Abscisic acid is tightly associated with changes in water availability to fine-tune plant growth [55,56,57,58,59,60] acting as a signaling molecule for plants to adjust their metabolism and growth in response to drought stress [55].

*A. thaliana* and *Camelina sativa* ABA-inducible WSD1 (Wax synthase/acyl-CoA:diacylglycerol acyltransferase) enhanced drought tolerance through leaf and stem wax loading and epicuticular wax accumulation [61]. Canola crop is sensitive to drought, which leads to severe yield losses. However, understanding the genetic basis of ABA-mediated drought tolerance will pave the way to engineering crops with improved drought resistance. Recently, the application of Omics approaches identified various ABA-induced and suppressed proteins involved in metabolism, photosynthesis, protein synthesis, membrane transport processes, protein folding/transport and degradation, and stress/defense responsiveness [62]. This finding suggests that various ABA-induced and suppressed metabolites were used as indicators in improving our knowledge of ABA signaling to drought tolerance.

### 3.3. Secondary Metabolites

**Figure 2:**
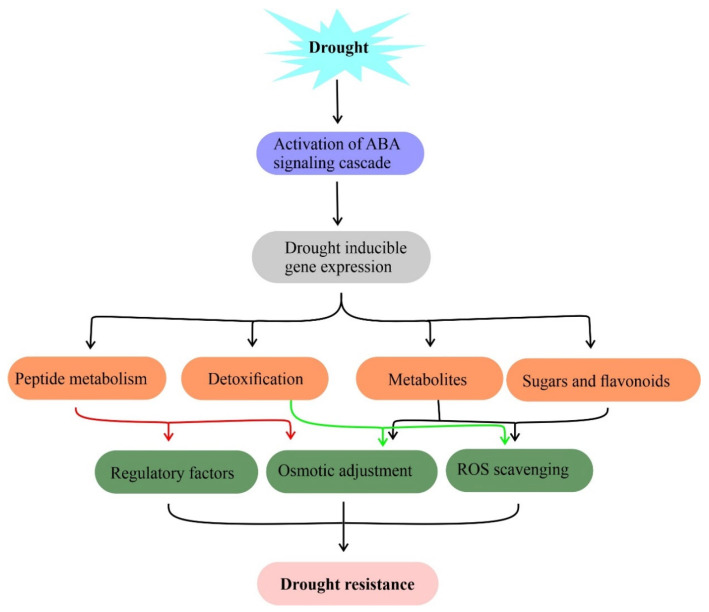
Metabolites and their functions in drought stress tolerance. Drought-induced accumulation of compatible solutes such as sugars, flavonoids, and amino acids for osmotic adjustment, free radical (ROS) scavenging to mitigate drought stress in plants. Genes involved in this metabolite biosynthesis against drought stress are useful in the metabolic engineering of drought resistance.

Plants are surrounded by a complex set of environmental stresses and respond equally to them. Plant metabolites are sensitive to changing environments such as drought [63]. The metabolic profiles of plants have been analyzed to predict their role under drought [64,65]. Plants have adapted two distinct strategies including osmotic adjustment [66] and accumulation of specialized secondary metabolites [67] to mitigate drought responses [68]. It has been shown that metabolites such as phenolic compounds, proline, glycine-betaine, soluble sugars, and other compatible solutes accumulated by plants during stress responses. These metabolites maintain water potential, cell turgor maintenance, osmotic adjustment, survival, stabilize proteins and membrane lipid bilayer structures under drought assisting to retain normal physiological processes (Figure 2) [66]. On the other hand, secondary metabolites act as scavengers of free radicals to mitigate oxidative stress in plants under drought stress.

Metabolic profiling revealed ABA-inducible metabolic networks in response to drought which encourages the accumulation of dehydration-inducible branched-chain amino acids, and key dehydration-inducible genes such as *lysine ketoglutarate reductase/saccharopine dehydrogenase (AtLKR/SDH), branch-chain aminotransferase (AtBCAT2), arginine decarboxylase,* and *delta 1-pyrroline-5-carboxylase (P5CS)* [69]. For instance, the accumulation of most amino acids such as tryptophan, glutamine, alanine, proline, aspartate, leucine, isoleucine, ornithine, valine, citric acid cycle precursors including cis-aconitate, succinate, and 2-oxoglutarate; flavonoids such as cyanidin and quercetin; and lipids such as acylated sterylglycosides and glycosyl inositol phosphoceramides were increased under drought in Arabidopsis [70,71,72] and a few crop plants, such as maize, barley, and rice [73,74,75]. In maize, *ZmPIS*, a phosphatidylinositol synthase, efficiently improved drought tolerance by altering membrane lipid composition and ABA biosynthesis [76]. Overexpression of *ABF3* in *Glycine max* significantly altered various primary and secondary metabolites such as glycerophospholipids, glycolipids, fatty acyls, prenol-lipids, and their derivatives [77].

## 4. Alteration in Root System under Drought

### 4.1. Root System Architecture

The plant root system anchors the plant into the soil and acquires water and minerals from the surrounding for the plant to develop and differentiate [78]. The primitive role of ABA is to modify root architecture, root growth pattern [79], and limit root growth [80], directly correlated with environmental perturbations [78], and in regulating lateral root emergence [81]. Genetic studies on ABA deficient mutants (*aba2-1* and *aba3-1*) exhibit an increased rate of lateral root emergence [82]. This was further evidenced by Gou et al. [83] who found that endogenous ABA biosynthesis requires inhibition of lateral roots in peanuts. Interestingly, ABA-insensitive (*abi4*) shows an enhanced number of lateral roots [84]. Therefore, instead of ABA levels, ABA signaling is also involved in lateral root formation.

Contrastingly, water stress regulates root cellular integrity and elongation by monitoring respiratory burst oxidase homolog (RBOH) gene expression via ROS secretions [85]. Although, molecular studies on *Medicago truncatula* mutants specified a significant role of ABA in maintaining root meristem [80]. Studies on ABA-deficient mutants have revealed that ABA is important to maintain root growth under drought [86]. Recently, Zhang et al. [87] concluded that ABA-mediated root growth of tomatoes under soil drying may involve auxin-dependent processes. ABA deficient mutants *vp5* and *vp14* develop stunt primary root in maize [88], but primary root elongation is resorted by exogenous IAA in tomato mutant notabilis (*not*) under drought [87]. However, ABA affects root architecture either positively or negatively based on the genotypic background and environmental conditions, offering a nuanced way to fine-tune the root system to compete with drought stress.

### 4.2. Root Secretions

**Figure 3:**
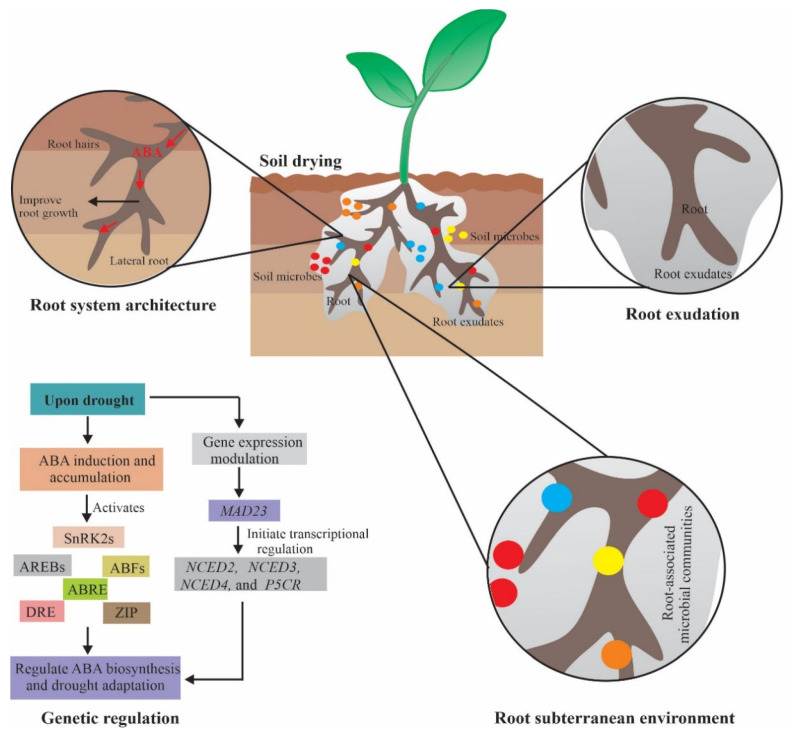
Role of ABA in mitigating drought tolerance at root system architecture that includes root exudates, microbial communities at the root-soil interface, and genetic and molecular regulation of various ABA-responsive genes and proteins. Endogenous ABA modulates the root system architecture by promoting root growth, soil microbial communities, and root exudation in response to soil drying. ABA accumulation upon drought led to the activation of TFs and modulates expression of genes responsible for improving ABA-mediated drought tolerance.

Root exudates are key drivers that play important role in responses to environmental perturbation [89] and serve as a nutritional or chemoattractant source with 11% to 40% of photosynthetically derived carbon [90,91]. Root exudates are enriched in organic acids, sugars, fatty acids, amino acids, and secondary metabolites [92], but the type and rate of exudation vary from species to species such as wild oat, grassland species, *Brassica napus*, and *A. thaliana* [93,94,95,96,97]. Plant exuded compounds assist in establishing diverse plant–microbial relationships [98] thereby enhancing plant ability to cope with environmental stresses (Figure 3) [99]. ABA acts as a mediator of drought via enhanced osmolytes biosynthesis including proline, organic acids, and protective proteins [100]. Yang et al. [101] demonstrated that ABA concentration was positively correlated with enhanced sucrose phosphate synthase activity and carbon remobilization. As expected, an increased endogenous root ABA concentration may enhance root exudates under water stress [102]. Garriga et al. [103] described that plant metabolites, more specifically ABA was significantly enhanced in root exudation upon drought stress [103]. These findings suggest the possible role of ABA in carbon remobilization and root exudation in mitigating plant stress tolerance (Figure 3).

Root exudates can adopt specific drought-resistant bacteria. Plant growth-promoting rhizobacteria (PGPR) encouraged the growth of wild-type plants and showed an opposite response in ABA-deficient mutant tomato [104]. Some bacterial species such as *Bacillus* [105] and *B. megaterium* [104] establish interaction with plant roots by endogenous ABA [105] promoting plant growth [106,107]. Henry et al. [108] reported that in maize organic exudation (including fumaric, malonic, succinic, and oxalic acids) increases under drought, which attracts *B. subtilis*, an important beneficial bacterium that enhances drought resistance of *Phleum pretense* via osmolytes secretion [109]. These findings suggest that endogenous ABA content is essential for promoting growth.

### 4.3. Root Subterranean Environment

The subterranean environment not only represents the dark region in the soil but also denotes variation in numerous abiotic stress such as temperature, water, and minerals availability. One of the major challenges in ecology is to identify and predict how different species respond to environmental perturbations [110]. Abscisic acid mediates root growth, rhizobacteria abundance, and influences root-hormonal status. For instance, Dodd et al. [111] reported that rhizobacteria can produce, metabolize, and utilize phytohormones/ phytohormonal precursors as a nutrient source. The hitherto study showed that rice seed inoculated with P6W and P1Y bacterial strains, ABA concentration was reduced to 14% by P6W and 22% by P1Y in the shoot, while root ABA concentration remain intact [112]. In the same study, tomato ABA-deficient mutants flacca (*flc*) notabilis (*not*) inoculated with P6W strains inhibited primary root extension and significantly increased biomass of WT plants [112].

Recently, Gowtham et al. [113] found that CRDT-EB-1 (*B. marisflavi*) extracted from the rhizosphere of mustard seedlings remarkably increased drought resistance by secreting ABA analog/xanthoxin. Additionally, a study by Porcel et al. [104] described that ABA-deficient mutants *flacca* and *sitiens* inoculated with *B. megaterium* showed restricted growth. This study concludes that ABA concentration is essential for *B. megaterium* growth, which promotes the growth of wild-type plants. Therefore, it may suggest that ABA-metabolizing rhizobacteria can modulate root-phytohormonal status and stimulate plant growth.

### 4.4. Biphasic Root Growth Responses

ABA is reported to either promote or inhibit root growth and is an inhibitor of plant shoot and root growth under well-watered conditions depending on its concentration. Mild soil drying stimulates root growth but inhibits root growth when it becomes more severe [44]. Similarly, ABA acts as an inhibitor of plant growth under water deficit. The biphasic effects of dry soil on root growth were mild, while water deficit stimulated root growth but severe water deficit inhibited root growth. Furthermore, complex biphasic effects of exogenous ABA on root growth under well-watered conditions were demonstrated by Li et al. [44]; low concentrations of ABA stimulated root growth while high concentrations inhibited root growth.

ABA also inhibits root growth in Arabidopsis by promoting ethylene biosynthesis and auxin influx [114]. In addition, ethylene insensitive mutants *etr1-1*, *ein2-1*, and *ein3-1*, auxin influx (*aux1-7*, *aux1-T*), and auxin-insensitive mutants *(iaa7/axr2-1)* unable to respond to the inhibitory effect of elevated ABA [44]. Furthermore, the stimulatory effect of low ABA concentrations was blocked by auxin efflux inhibitors (*pin2/eir1-1)* but less pronounced in auxin efflux mutant (*iaa7/axr2-1*). Taken together, ABA may play a pivotal role in biphasic root growth in plants.

## 5. Molecular Mechanism of ABA-Mediated Drought Regulation

ABA regulates different physiological and molecular responses under drought. Plants perceive stress stimulus in their roots and leaves and transmit the signal to their shoots to synthesize ABA. Protein kinases are positive regulators of ABA signaling, metabolism, and transport in response to drought [115]. For instance, an ABA-responsive G protein receptor (GCR2) caused insensitivity in germination and influenced the expression of ABA inducible genes [116]. Moreover, ABA has been reported to interact with the flowering-time control protein (FCA) and the Mg-chelatase H subunit [117]. Sensors sense drought stimuli on the membrane, and these signals are then passed down through multiple signal transduction pathways, resulting in the expression of drought-responsive genes. Recently, several classes of ABA biosynthesis *NCED1*, *NCED3*, *ABA2*, and *AAO3* and transporter proteins AtABCG25 and AtABCG40 localizing in the plasma membrane indicated that plants possess a complex system to sense and respond to fluctuating environmental conditions [118]. Nitrate transporter 1/peptide transporter protein (NPF) named AIT1 initially, mediates ABA uptake into cells, suggesting that this protein could also have a role in intercellular ABA transport [119].

To gain an insight into the genetic regulation of the root system under drought stress, several studies have been investigated. Recently, Zhang et al. [120] reported transcriptomic analysis at three time scales (1 h, 3 h, and 7 h) under drought in *Pearl millet*. It was observed that a total of 2004, 1538, and 605 genes were differentially expressed at 1 h, 3 h, and 7 h, respectively, while 12 genes were upregulated at all the time scales. Some of these highly expressed genes were related to the MAPK signaling pathway, metabolic processes, and plant hormonal signaling such as the ABA signal transduction pathway. This may provide a genetic basis to understand drought resistance mechanisms in other plants. Similarly, Li et al. [121] showed that *OsMAD23* serves as a positive regulator for altering ABA biosynthesis to enhance drought tolerance in rice. *OsMAD23* encourages endogenous ABA accumulation through its biosynthesis and proline accumulation by activating several ABA and proline biosynthesis genes including *OsNCED2/3/4* and *OsP5CR*. These findings suggest a new way to enhance drought/salt resistance in rice.

The expression of several TFs is controlled by ABA such as AREBs, ABFs, DRE, ABRE, and several others (Figure 3). Indeed, transactivation analysis revealed that the ABA-responsive TF factor *OsBZIP72* activates the expression of *OsSWEET13* and *OsSWEET15* by binding with their promoters mediating sucrose transportation and distribution in rice under drought and other stresses [122]. It has also been found that Arabidopsis *ZmbZIP33* remarkably improved chlorophyll content and root length as a drought-adapted strategy in maize [123]. A few popular studies demonstrating the role of ABA-mediated drought tolerance are listed in Table 1.

### ABA Dependent Translational and Posttranslational Modification

Post-transcriptional regulation such as alternative splicing and RNA-mediated silencing, and post-translational regulation such as protein activity, subcellular localization, and protein half-life significantly contribute to the fine-tuning of ABA-dependent plant response drought stress.

Post-transcriptional regulation of an ABA-responsive basic leucine zipper (bZIP) TF ABI5 has been studied extensively in response to various stresses, particularly drought. For instance, ABI5 represses/activates the expression of various ABA-dependent receptors and kinases and stimulates plant adaptation to drought stress [149,150]. Several members of the bZIP family (ABF1-4) were shown to be highly redundant to ensure ABA-mediated adaptation to drought in various species such as in *Arabidopsis*, rice, cotton, carrot, and barley [151,152,153,154,155]. ABI5 interaction is activated through phosphorylation by kinases SRK2D/SnRK2.2, SRK2E/SnRK2.6/OST1, SRK2I/SnRK2.3, SOS2-like protein kinase 5/CIPK11, glycogen synthase kinase 3-like kinase BRASSINOSTEROID INSENSITIVE 2 (BIN2) and calcineurin B-like interacting protein kinase 26 (CIPK26), which suppress or activate ABI5 post-translation under ABA treatment. Therefore, ABI5 protein regulation could serve as a model to study the activity and stability of critical components engaged in drought tolerance affecting posttranslational modification. Various key components in drought and ABA signaling under post-translational modifications result in modulation of drought responses indicating their role in stress adaptation (Table 2).

## 6. Hormone Crosstalk

In plants, hormones secreted from predominantly vascular cells and guard cells and transported to distant target sites through the plant body circulatory system, suggesting that most plant hormones are mobile. When plants are exposed to environmental stress the ABA signaling cascade is rapidly activated, which in return activates ABA-responsive TFs and induces the expression of ABA-responsive genes [166]. ABA interacts with other hormones including auxin, gibberellins (GA), cytokinin (CK), ethylene (ET), salicylic acid (SA), and jasmonic acid (JA) to help the plant to withstand abiotic stresses such as drought. Phytohormonal engineering is a major environmental stress mediator, particularly ABA, a prime regulator of environmental stress tolerance. ABA is produced in shoot and roots but enhanced accumulation is stimulated by a decrease in cellular turgor [167]. However, ABA accumulation is directly correlated with plant/tissue water status, but the underlying molecular mechanism of ABA accumulation and drought sensation is still unclear.

Stress response in plants is regulated by integrating ABA and auxin signals [168]. Some studies revealed that ABA and auxin distribution within the primary roots and lateral roots are independent of each other because they show different localization patterns. Furthermore, auxin biosynthesis was inhibited by ABA, but ABA accumulation modulates auxin transport in the root tip of Arabidopsis during drought [169,170]. Some authors argue that auxin and ET are required for ABA response in the root [171]. Furthermore, auxin/ET/ABA crosstalk was examined, and it was found that auxin and ET likely operate in a linear pathway to affect ABA-responsive inhibition of root elongation, and probably act independently to affect ABA-responsive inhibition of seed germination [171]. Exogenous application of JA encourages foliar ABA accumulation while JA deficiency suppresses ABA levels [172,173], suggesting extensive crosstalk between JA–ABA pathways. Puértolas et al. [174] demonstrated that ABA concentration increase with declining plant water status while JA accumulates during early stages of stress, however, the molecular and physiological involvement of JA in concert with ABA is still unclear.

Abscisic acid enables plants to cope with several abiotic stresses by interacting with important ABA-related TFs and by coordinating with other plant hormones. For instance, ABA-related DREB (dehydration-responsive element binding), nced (9-cis-epoxycarotenoid dioxygenases), and MYB (myeloblastosis) TFs are known to regulate intracellular pathways involved in CK homeostasis [175]. AtMYB2 knockout led to enhanced expression of IPT1/4/5/6/8 (adenosine phosphate-isopentenyl transferases), suggesting the role of AtMYB2 in CK synthesis [175]. Similarly, overexpression of MsDREB6.2 (dehydration-responsive gene) resulted in increased *MdCKX4a* expression, thereby reducing endogenous CK and enhancing drought tolerance of transgenic apple plants [176].

**Figure 4:**
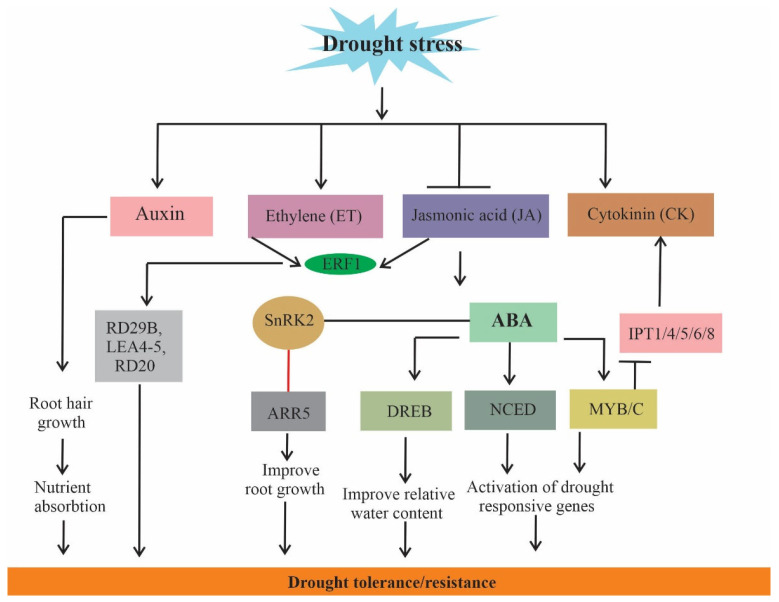
Proposed model of auxin, ethylene (ET), jasmonic acid (JA), cytokinin (CK), and ABA cross talk under drought stress. ERF1 (ETHYLENE RESPONSE FACTOR1) modulates expression of potential genes involved in drought tolerance. ABA-responsive component SnRK2, directly phosphorylates type-A RR5 (ARR5), ABA activates several transcription factors (TFs) which could result in enhanced drought tolerance.

In *Arabidopsis*, the SnRK2 protein interacts with CK signaling-ARR5 (type-A RR5) and regulates ABA-mediated drought tolerance [177]. In addition, ET crosstalk with ABA in an antagonistic manner via regulating root and shoot growth under drought [178]. Upon abiotic stress, along with ROS such as drought, inositol phosphate is produced, resulting in enhanced endogenous ABA levels [179]. Crosstalk between ABA and SA assists plant-water budget, osmotic adjustment, stomatal conductance, distribution of photoassimilates, and leaf senescence [178]. The expression of ERF1 (ethylene response factor1) is rapidly induced by ET and JA individually or synergistically [180]. Overexpression of ERF1 encourages drought tolerance and increases the accumulation of ABA levels and Pro, which restrict water loss and significantly contribute to stress resistance [181]. ERF1 activates the expression of LEA4-5 (late-embryogenesis abundant protein4-5) thereby resulting in drought tolerance (Figure 4) [182].

## 7. Other Related Mechanisms

Several studies on drought have shown that the ABA hormone has a significant role in regulating the biochemical mechanisms that enable drought-prone plants to rapidly grow, flower, and produce mature seeds just before the onset of drought. Hwang et al. [183] confirmed that ABA plays an important role in regulating flowering during accelerated floral transition in *A. thaliana.* The study further showed that ABA-binding factors, ABF3 and ABF4 in *A. thaliana* enhance flowering by inducing SOC1 transcription, thus enabling the plant to complete its lifecycle under drought stress [183].

Besides ABA hormone responses, plants possess other ABA-independent mechanisms through which they mitigate drought effects [184]. The role of nutritional stress in drought stress alleviation remains a key area of focus. Drought normally results in depleted nutrients which lead to poor plant growth and development, and finally low crop yields. Studies have shown that plants associated with high nutrient use efficiency can ameliorate stress effects and cope with drought [185,186]. Micro and macronutrients including phosphorus [187], calcium [188], potassium [189], nitrogen [190], silicon [191], magnesium, and zinc [192] were reported to help in alleviating the adverse effects of drought. Although several studies have shown how different micro and macronutrients in plants are affected by drought, more research is needed to decipher the interactive mechanisms of these molecules with plants.

**Figure 5:**
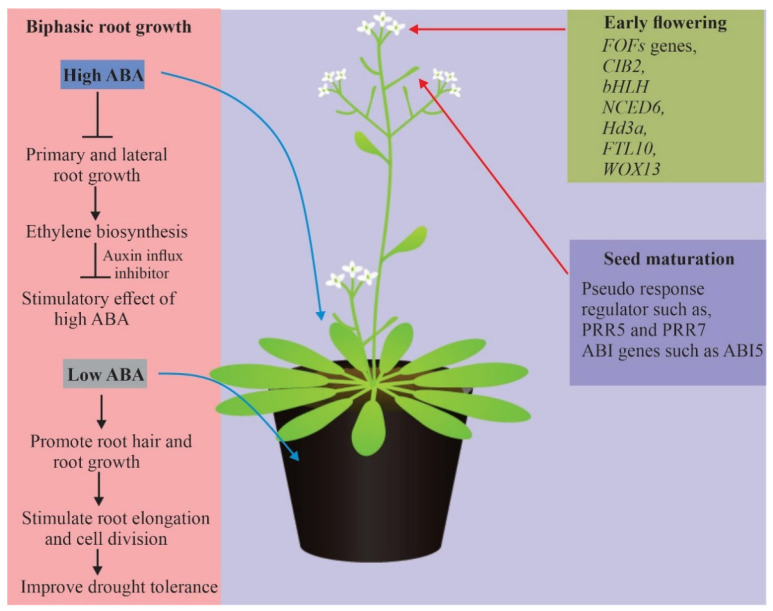
The molecular basis of ABA-mediated plant response to drought. A high concentration of ABA imposed a negative effect on auxin influx, while a low concentration of ABA promotes root growth resulted in enhanced drought tolerance.

ABA-deficient mutant rice, however, induced low ABA and delayed flowering. The study reported some of the light receptors, circadian components, and genes related to flowering including *OsTOC_1_, Ghd*_7_, and *PhyB* involved in drought stress in an ABA-dependent manner. Several studies [183,193,194] have also reported ABA to modulate drought escape by activating the expression of florigen genes FT and TSP, and the floral integrator *SOC_1_* (ABF_3_ and ABF_4_) in *Arabidopsis*. Understanding the connection between ABA and drought escape is of importance in practical circumstances whereby crops are under irrigation, in such a scenario, the ABA responses could be targeted to ensure maximum crop production. The knowledge tapped from studies on drought escape mechanisms in plants needs to be utilized in developing drought-resistant crops that can easily withstand the dry seasons thus enhancing food production for the increasing world population (Figure 5).

Drought—at the early stage of plant development—could trigger early flowering and reduce tiller numbers by inducing the accumulation of ABA [195] by activating ABA signaling components that control water status and stomatal closure, promoting plant escape or adaptation to drought stress. Similarly, trehalose treatment upregulates ABA signaling-related gene expression via activating the ABA signaling pathway, regulating stomatal aperture, and preventing transpirational water loss [196]. It was reported that drought has feedback effects on the circadian clock by simultaneously regulating many flowering-related genes such as *OsTOC1*, *Ghd7*, *OsGI*, *OsELF3*, *OsPRR37*, *OsMADS50,* and *PhyB,* which promotes early flowering [195,197] in an ABA-dependent manner [195].

## 8. Conclusions and Perspective

Environmental stresses, particularly drought, impair plant growth and productivity threatening global food security. Therefore, the development of drought-resistant crops is an important implication to sustainable crop production. Plants have evolved a series of adaptive strategies at cellular and molecular levels to cope with environmental cues including drought. The underlying genetic and molecular mechanisms of drought tolerance and escape are still an emerging topic in plant biology. Drought escape is the adaptive mechanism through which plants undergo rapid development to complete their life cycle before the onset of serious water deficits [198]. ABA is a key hormone and critical regulator of different stresses, including drought and salinity in various plant species. The overall progress of research on ABA-mediated drought responses has revealed its key role in growth and development during stress conditions. However, how plants perceive and transmit drought stress to different cells to initiate ABA accumulation for drought resistance remains unclear.

ABA receptors are present in almost every tissue and expressed specifically in each tissue allowing the plant to sense environmental signals and transmit them accurately to the target tissues. ABA regulates many aspects of plant growth, development, regulation of stomatal closure, channel activities in guard cells, promoting proline synthesis and accumulation, transcription of calmodulin protein, accelerated floral transition and seed maturation, and expression of ABA-drought responsive genes [199,200,201]. To achieve this, various drought-responsive genes and metabolic pathways are triggered leading to beneficial adjustments in the morphological structures and growth rate [86].

Various regulatory factors complement cell-to-cell or tissue-to-tissue communication and long-distance translocation from tissues for synthesis to target tissues. Recently, advances have been made in understanding the ABA regulatory mechanism at the molecular and cellular levels providing novel insights for biotechnology and agriculture merits for further research for crop breeding programs and crop innovation. Moreover, integrated phenomics will be key in addition to other high-throughput approaches to assess plant phenotype against drought stress. Similarly, artificial intelligence and machine learning are powerful techniques to understand these complex integrated systems in plant responses to drought.

## Figures and Tables

**Table 1:** Some important genes or transporters for improved drought tolerance through modulating ABA signaling pathway in plants.

Plant Species	Gene/Transporter	Function	References
*Agrostis* grass	*VuNCED1*	Increased 3–4-fold plant biomass	[124]
*Arabidopsis thaliana*	*AtABCG17*-*18*	Stomatal conductance, and increased water use efficiency	[125]
	*AtPDR12/ABCG40*	ABA uptake transporter	[32]
	*AIT1/NRT1.2*	ABA importer important for stomatal aperture	[126]
	*AtBBD1*	Increased expression of ABA and drought-responsive genes	[127]
	*AtSAUR32*	Highly induced by abscisic acid and drought treatment	[128]
	*AtbHLH68*	Lateral root elongation in response to drought	[129]
	*AtHDA9* and *ABI4*	Regulate *CYP707A1* and *CYP707A2* expression under drought	[130]
	*AtDTX/MATE*	Facilitate ABA efflux and tolerance to drought	[15]
*Brachypodium distachyon*	*BdABCG25*	Regulate intercellular ABA transport	[131]
*Brassica napus*	*BnFTA*	Improved under drought conditions	[132]
*Glycine max*	*GmCIPK2*	ABA signaling and drought tolerance	[133]
*Nicotiana tabacum*	*SgNCED1*	Enhanced ABA accumulation increased drought tolerance	[134]
*Oryza sativa*	Oshox22	Increased ABA content, and enhanced drought tolerance	[135]
	*OsbZIP46CA1*	Improved drought resistance	[136]
	*OsPM1*	ABA influx carrier is important in drought responses	[137]
*Petunia*	*LeNCED1*	Elevated levels of ABA and proline, increases drought resistance	[138]
*Setaria italica*	*SiARDP* target of *SiAREB*	ABA-dependent signal pathways	[139]
*Solanum lycopersicum*	*SlGRAS42s*	ABA signaling	[140]
	*PYR/PYL/RCAR*	Role in seed germination and basal ABA signaling	[141]
*Triticum aestivum*	*HVA1*	Improved growth characteristics under water deficit	[142]
*Vigna unguiculata*	*VuABCG25*	Involved in ABA signaling pathway under water stress	[143]
*Vitis vinifera*	*VviNCED1*, *VviNCED2*	ABA synthesis in response to plant water status	[144,145,146]
*Xanthoceras sorbifolium*	*XsWRKY20*	Regulate drought tolerance by ABA signaling pathway	[147]
*Zea mays*	*ZmXerico1-2*	Improved water use efficiency, yield under drought stress	[148]

**Table 2:** Post-translational modifications and their predicted role in plants responsive to drought and ABA signaling in different plant species.

Plant Species	Protein	Target	Role in Plants	References
Arabidopsis	PUB22/23	RPN12a	Drought tolerance and ABA signaling	[156]
Arabidopsis	PUB19	nd	Drought tolerance	[157]
Arabidopsis	AIRP1	nd	ABA-dependent drought tolerance	[158]
Arabidopsis	Rma1	PIP2;1	Drought tolerance	[159,160]
Arabidopsis	SDIR1	SDIRIP1	Drought and salinity tolerance, ABA signaling	[161]
Arabidopsis	XERICO	nd	Drought stress tolerance, ABA biosynthesis	[162]
Arabidopsis	DOR	nd	Drought stress tolerance	[163]
Rice	RING-1	nd	Drought tolerance and ABA response	[164]
Maize	ZF1	nd	Drought tolerance and ABA signaling	[165]

## Data Availability

All data were available within the manuscript.
